# Type 1a Endoleak after Fenestrated Frozen Elephant Trunk Technique

**DOI:** 10.3400/avd.cr.23-00074

**Published:** 2024-02-09

**Authors:** Yoshitaka Yamane, Tomokuni Furukawa

**Affiliations:** 1 Department of Cardiovascular Surgery, Cardiovascular Center, Akane-Foundation Tsuchiya General Hospital, Hiroshima, Hiroshima, Japan

**Keywords:** aortic dissection, frozen elephant trunk, thoracic endovascular aortic repair

## Abstract

The patient was a 48-year-old man who had undergone fenestrated frozen elephant trunk (FET) technique for acute type A aortic dissection. Postoperative enhanced computed tomography (CT) imaging revealed a type 1a endoleak from the fenestration. Nevertheless, the stented portion of the FET exhibited circular expansion. However, 2 months after surgery, enhanced CT imaging revealed the flattening of the FET due to the persistent endoleak and we performed an urgent zone 2 thoracic endovascular aortic repair (TEVAR). When type 1a endoleak from the fenestration is observed, the FET can be flattened, as in this patient, additional intervention should be considered.

## Introduction

Recently, favorable surgical outcomes of the fenestrated frozen elephant trunk (FET) technique have been reported.^1)^ However, the fenestrated FET procedure has led to some complications, including endoleak, and their incidence and associated events remain unknown. We report a patient who underwent the fenestrated FET technique and in whom an endoleak from the fenestration resulted in the collapse of the FET that necessitated subsequent thoracic endovascular aortic repair (TEVAR).

## Case Report

A 48-year-old man was admitted to our institution due to the sudden onset of severe back pain. Enhanced computed tomography (CT) imaging revealed an acute type-A aortic dissection with a thrombosed false lumen, in which the entry tear was localized in the descending thoracic aorta.

We promptly executed an emergent total arch replacement (TAR) with a four-branched J graft (Japan Lifeline, Tokyo, Japan) utilizing the fenestrated FET technique. A comprehensive description of our FET technique is available in a previous publication.^2)^ The procedure commenced with establishing a total cardiopulmonary bypass (CPB) via ascending aortic and bicaval cannulation. Selective cerebral perfusion was initiated upon achieving a rectal temperature of 28°C. Subsequently, a longitudinal incision was made in the aorta, proximal to the left subclavian artery (LSCA). We used Frozenix (Japan Lifeline), which was a commercially available Japan-made FET prosthesis released in 2014. The FET (Frozenix, FRZX-25120) was carefully inserted into the true lumen of the descending aorta. A 10-mm fenestration was created within the nonstented segment of the Frozenix at a location corresponding to the origin of the LSCA. This fenestration was fortified externally with U-shaped felt. An artificial graft featuring four branches was anastomosed end-to-end at the aortic arch stump, encompassing both the aortic wall and the FET. Further reconstruction encompassed the left common carotid artery, brachiocephalic artery, and the ascending aorta.

**Figure 1:**
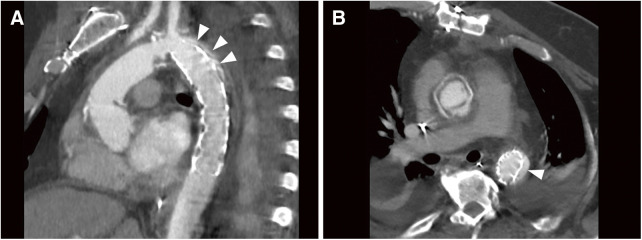
Fig. 1 Enhanced CT imaging (**A**, **B**) after the fenestrated FET technique shows antegrade blood flow originating from the fenestration in the FET (arrowheads). The stented portion of the FET shows a circular expansion. CT: computed tomography; FET: frozen elephant trunk

Subsequent postoperative enhanced CT imaging revealed an antegrade blood flow originating from the fenestration in the FET (type 1a endoleak), directed toward the true lumen of the distal aortic arch (**Fig. 1**). Nevertheless, the stented portion of the FET exhibited circular expansion. Given this, a decision was made to adopt a observation without reintervention regarding the endoleak, and the patient was discharged 1 month after surgery.

**Figure 2:**
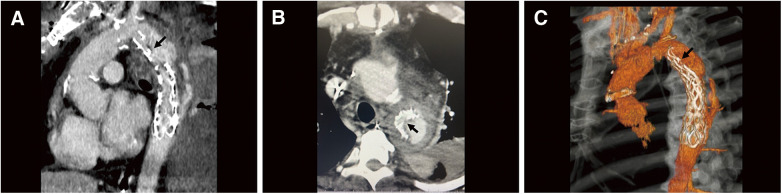
Fig. 2 Enhanced CT imaging (**A**, **B**, **C**) 2 months after surgery shows flattening of the FET as a consequence of the persistent endoleak (black arrow). CT: computed tomography; FET: frozen elephant trunk

**Figure 3:**
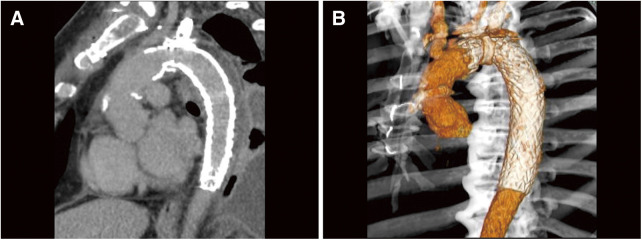
Fig. 3 Enhanced CT imaging (**A**, **B**) after TEVAR, revealing the absence of an endoleak and the good expansion of the stent graft. CT: computed tomography; TEVAR: thoracic endovascular aortic repair

However, the patient was readmitted to our facility 2 months after surgery due to a sensation of coldness in the lower extremities. Subsequent enhanced CT imaging revealed the flattening of the FET due to the persistent endoleak (**Figs. 2A**–**2C**). Consequently, an urgent TEVAR was undertaken, coupled with coil embolization of the LSCA to encapsulate the fenestration. Deployment of a stent graft (Gore TAG, W. L. Gore & Associates, Inc., Flagstaff, AZ, USA) in the zone 2 position resulted in the subsequent resolution of the endoleak. We did not conduct LSCA reconstruction since we had verified the LSCA network before initial surgery. The patient’s postoperative course transpired without noteworthy incidents, and the follow-up enhanced CT imaging revealed the absence of an endoleak and the good expansion of the stent graft (**Figs. 3A** and **3B**).

Institutional review board approval was obtained for the fenestrated FET technique. Consent from the patient was obtained for this case report.

## Discussion

Fenestrated FET is a method of reconstruction that obviates the need for anastomosis of the neck vessels during TAR. Its utility has been documented previously in the medical literature.^1^^,^^3)^ Recently, early and midterm outcomes of the fenestrated FET technique for acute type-A aortic dissection have been reported, demonstrating excellent initial results without any fenestration-related complications, including fenestration site occlusion or endoleak-related aortic events.^3)^ The authors noted that simply performing fenestration of the FET could result in a minor endoleak between the aortic intima and the FET, a concern they addressed by fixing the fenestration site to prevent an endoleak. They performed fixation of the FET using continuous 4-0 polypropylene sutures in close proximity to the fenestration to prevent endoleak through the fenestration.

Okugi et al. detailed their experience employing the fenestrated FET technique in a patient with an isolated left vertebral artery.^4)^ However, a type 2 endoleak originating from the left vertebral artery and a type 1a endoleak stemming from the fenestration were identified. Consequently, additional TEVAR and coil embolization of the left vertebral artery were performed.

No anatomical anomalies were noted in the present case, and the fenestration site was fixed. Nonetheless, postoperative enhanced CT imaging revealed an endoleak emanating from the fenestration. Initially, the FET exhibited a circular expansion; however 2 months later, it assumed a flattened configuration. We postulate that the endoleak from the fenestration gradually intensified, surpassing the radial force exerted by the FET. In this instance, we posit that one of the primary contributors to flattening of the FET was the creation of the fenestration in a non-stented region. To mitigate this, fenestrations should be strategically positioned within the stent segment of the FET, coupled with robust reinforcement to avert endoleak occurrence.

Endoleak management resulting from fenestration primarily involves covering the fenestration using TEVAR and embolization of the LSCA.

While recognizing the potential of fenestrated FET as a productive approach for treating acute type-A aortic dissections, the issue of endoleak occurrence remains unresolved. Okamura et al. mentioned that uncertainty persists regarding whether an endoleak into the true lumen can lead to aortic events.^3)^

In this case, we followed up with the patient, knowing that there was type 1a endoleak. Nevertheless, this decision proved to be wrong and the patient required emergency additional intervention. In view of this case, we think that once type 1a endoleak is detected, observation is unwarranted and we should not hesitate to perform additional intervention.

## Conclusion

We report a patient who underwent the fenestrated FET technique and in whom type 1a endoleak from the fenestration resulted in the collapse of the FET that necessitated subsequent TEVAR. When a type 1a endoleak from the fenestration is observed, the FET can be flattened, as in this patient, additional intervention should be considered.

## Disclosure Statement

No conflict of interest is declared.

## Author Contributions

Study conception: YY

Writing: YY

Critical review and revision: all authors

Final approval of the article: all authors

Accountability for all aspects of the work: all authors.
